# The Role of Weight Suppression in Intensive Enhanced Cognitive Behavioral Therapy for Adolescents with Anorexia Nervosa: A Longitudinal Study

**DOI:** 10.3390/ijerph20043221

**Published:** 2023-02-12

**Authors:** Simona Calugi, Anna Dalle Grave, Maddalena Conti, Laura Dametti, Mirko Chimini, Riccardo Dalle Grave

**Affiliations:** Department of Eating and Weight Disorders, Villa Garda Hospital, Via Monte Baldo 89, 37016 Garda, VR, Italy

**Keywords:** adolescents, inpatient treatment, cognitive behavioural therapy, treatment outcomes, weight suppression, anorexia nervosa, BMI

## Abstract

The study aimed to establish the role of weight suppression in a cohort of adolescents with anorexia nervosa treated with intensive enhanced cognitive behavioral therapy (CBT-E). One hundred and twenty-eight adolescent patients with anorexia nervosa (128 females and 2 males), aged between 14 and 19 years, were recruited from consecutive referrals to a community-based eating disorder clinic offering intensive CBT-E. Weight, height, Eating Disorder Examination Questionnaire, and Brief Symptom Inventory scores were recorded at admission, end-of-treatment, and at a 20-week follow-up. In addition, the developmental weight suppression (DWS, difference between one’s highest premorbid and current z-BMI, i.e., BMI z-scores) was calculated. The mean baseline z-BMI was −4.01 (SD = 2.27), and the mean DWS was 4.2 (SD = 2.3). One hundred and seven patients (83.4%) completed the treatment and showed both considerable weight gain and reduced scores for eating-disorder and general psychopathology. Among completers, 72.9% completed the 20-week follow-up and maintained the improvement reached at the end-of-treatment. DWS was negatively correlated with end-of-treatment and follow-up z-BMI. This indicates that weight suppression is a predictor of the BMI outcome of intensive CBT-E and confirms that this treatment is promising for adolescents with anorexia nervosa.

## 1. Introduction

Weight suppression is the discrepancy between an individuals’ highest past weight at adult height and their current weight. Most of the literature on this measure has been published in the last decade, and demonstrates associations with several measures of eating pathology, weight change, hormone levels, eating disorder (ED) prognosis, and ED treatment outcome [1,2].

Most studies into this measure have focused on bulimia nervosa, finding that greater weight suppression is associated with increased eating disorder psychopathology, increased frequencies of binge-eating and purging episodes, and increased weight gain during treatment in such patients [1,2]. To date, only few studies have examined weight suppression and treatment outcomes in anorexia nervosa, mainly in adults. These have revealed correlations between weight suppression at admission and greater [3,4,5,6] and more rapid weight gain during treatment [3,4,6]; weight suppression at the time of discharge from acute treatment, on the other hand, has been linked to the change in weight over time. In particular, individuals with greater weight suppression were found to be less likely to lose weight over the following year [7]. However, this contrasts with the results of a recent study, which found that weight suppression does not substantially impact the likelihood of successful weight maintenance or time-to-relapse following restoration to a minimally normal weight in adult outpatients with anorexia nervosa [8]. Nonetheless, several studies have suggested an association between the degree of weight suppression, or its interaction with body mass index (BMI), and eating disorder symptomatology [3,6,7].

Weight suppression has been little studied in adolescents with anorexia nervosa. In a naturalistic study on a community sample of adolescent-onset anorexia nervosa, Witt et al. [9] found that higher weight suppression at the time of the lowest weight was associated with a higher BMI at 6- and 10-year follow-ups. In addition, a retrospective cohort study on adolescent patients with anorexia nervosa found that binge-eating behavior, increased speed of weight loss, and total weight loss during the course of anorexia nervosa were significantly more likely in patients with a history of excess weight, as compared to patients with normal premorbid weight [10]. Finally, a prospective longitudinal study in 201 adolescents with a restrictive eating disorder and a wide range of BMIs at presentation found that lower weight suppression at follow-up was associated with a favorable one-year outcome of family-based treatment (as defined by the absence of clinically significant eating-disorder psychopathology) [11].

However, no study has yet investigated the role of weight suppression in adolescent patients with anorexia nervosa treated with enhanced cognitive behavioral therapy (CBT-E) to assess whether weight suppression could represent a predictor of treatment outcome.

## 2. Materials and Methods

### 2.1. Design

In this longitudinal observational study, the sample consisted of a cohort of adolescents (aged 14–19 years) seeking treatment for anorexia nervosa at a specialist eating-disorder unit in Garda-Verona (northern Italy) affiliated with the Italian National Health System. They were consecutively recruited, from September 2015 to December 2021, and screened for eligibility for an intensive real-world 20-week CBT-E program. The program consisted of 13 weeks of treatment as inpatients, followed by 7 weeks in a day-hospital setting. The data were collected at baseline, at the end of the 20-week treatment (EOT), and 20 weeks later.

Each patient provided informed written consent to their clinical data being collected and processed anonymously. The parent(s) and/or legal guardian(s) of those under the age of 18 provided informed consent on their behalf. The research was conducted in a service-level setting, with all involved procedures being performed as part of routine clinical practice. The study was conducted in accordance with the Declaration of Helsinki, and the protocol was approved by the GHC Institutional Review Board (Protocol Code 0001GHCIRB).

### 2.2. Participants

Participants were referred to the clinic by their general practitioner or a secondary healthcare professional. The following inclusion criteria were applied: (a) from 14 to 19 years of age; (b) meeting the Diagnostic and Statistical Manual of Mental Disorders, edition 5 (DSM-5) diagnostic criteria for anorexia nervosa [12] in assessment sessions conducted by an eating disorder specialist; and (c) previous failure of outpatient treatment for anorexia nervosa (either because they lost weight or gained insufficient weight, or showed little reduction in unhealthy weight control behaviors and/or binge-eating episodes after at least 8 weeks of treatment). No patients with acute psychotic disorder or concomitant substance use disorder were included. Overall, 128 patients were included in the study.

### 2.3. The Treatment

Intensive CBT-E has been described in great detail in previous publications [13,14].

Based on the disease model, CBT-E involves progressive patient education and the implementation of gradual behavioral changes. Patients then work with the therapist to analyze the implications of these changes and to learn problem-solving skills and other cognitive behavioral strategies to help them overcome any obstacles to their progress. Admission to the intensive CBT-E unit is voluntary. This approach is used because, according to cognitive behavioral theory, actively involving patients in decision-making is used to enhance their sense of empowerment and control, a fundamental factor in their ability to overcome their eating disorders.

Before starting the treatment, therefore, three to four preparatory sessions are held in which the prospective patient is helped (rather than coerced or instructed) to decide to start the program, which involves addressing weight restoration from day one. Their treatment is overseen and administered by a dedicated team of healthcare professionals, all trained in and ascribing to the ethos of CBT-E. They will attend one-to-one sessions with a CBT-E-trained clinical psychologist twice weekly in the first four weeks, and after that once a week. CBT-E-based group sessions are also held four times a week. During meals, a CBT-E dietitian will be on hand to assist them until they achieve a BMI of ≥18.5. A CBT-E-trained physician will oversee their physical health, and CBT-E-trained nursing staff will supervise any medications required and assist them with implementing treatment and managing any difficulties. The unit is open, so patients can learn to cope with environmental triggers with a dedicated team of staff on hand, and school-age patients are expected to continue their studies, with accredited teachers being provided by the unit. 

Otherwise, the treatment is similar to outpatient CBT-E [14,15]. Once the cognitive behavioral mechanisms reinforcing their eating disorder have been disrupted and the patients have reached a low healthy weight, the focus of CBT-E shifts to relapse-prevention. As such, patients work on recognizing the early warning signs that their “eating-disorder mindset” is re-emerging; they learn techniques that will enable them to prevent it reactivating. During this phase, they will also work on identifying likely environmental setback triggers and learning how to deal with them effectively using CBT-E procedures. Parents and/or other significant others are recruited at this stage to help facilitate their transition and manage stressful times at home.

Once the intensive CBT-E program has ended, patients are invited to attend 20 “optional” sessions of post-inpatient outpatient treatment. These are held twice weekly in the first month, once weekly in the second and third months, and then once every fortnight in the fourth and fifth months. These sessions are focused on developing relapse-prevention skills and addressing any obstacles and/or residual eating-disorder features through CBT-E strategies and procedures.

### 2.4. Assessment

The following measures were used to assess participants at baseline, EOT, and at a 20-week follow-up:

- Body weight and BMI: BMI-for-age percentiles were calculated using the Center for Disease Control and Prevention growth charts [16]. (http://www.cdc.gov/growthcharts/percentile_data_files.htm (accessed on 5 May 2022)). To this end, each patient’s weight was measured on a beam balance scale, and their height on a wall-mounted stadiometer. A BMI-for-age percentile with a value < 1 was calculated as 0.5.
- Weight suppression: following Singh et al.’s [17] suggestions, each patient’s developmental weight suppression (DWS) was calculated as the difference between their highest premorbid z-BMI (i.e., BMI z-score) and their current z-BMI. The z-BMIs were calculated using the mean value expected for the patient’s age and standard deviation, via the Pediatric Z-Score Calculator (Children’s Hospital of Philadelphia).
- Eating-disorder features were assessed via the Eating Disorder Examination Questionnaire (EDE-Q, 6th edition, Italian version) [18,19]. The EDE-Q assesses the eating disorder psychopathology over the last 28 days. It includes four subscales (restraint, eating concern, weight concern, and shape concern) and a global score. The internal consistency in our sample was 0.96.
- General psychiatric features were assessed via the Brief Symptom Inventory (BSI, Italian version) [20,21]. The BSI assesses the clinical symptoms as indicators of emotional distress in the last 7 days. In our sample, the internal consistency was 0.97.
- Functional impairment secondary to the eating disorder was assessed via the Italian version of the Clinical Impairment Assessment (CIA) [22,23]. The CIA refers to last 28 days and the global score refers to the clinical impairment secondary to the eating disorder. The internal consistency in our sample was 0.95.

### 2.5. Outcome Categories

There were two operational outcome categories adopted. The first, termed “good BMI outcome” was taken as the patient reaching the lowest threshold in a healthy BMI range [24], namely a BMI percentile corresponding to an adult BMI of ≥18.5 kg/m^2^ [25]. The second, termed a “full response”, was when their global EDE-Q score fell below 1 SD above the community mean (i.e., <2.77) [26] and they reached a BMI-for-age percentile corresponding to an adult BMI of ≥18.5 kg/m^2^—considered a simple, replicable way of defining an excellent outcome [27].

### 2.6. Statistical Analysis

As for the descriptive statistics and comparisons, continuous variables are reported as means and standard deviations (SD), and categorical variables as percentages. Missing data in z-BMI, EDE-Q global and subscale scores, and BSI and CIA global scores at EOT and follow-up, was considered arbitrary, and was therefore handled via the iterative Markov-chain Monte Carlo method. The fully conditional specification method of this multiple imputation procedure was applied, alongside predictive mean matching [28]. “Missingness” (0/1) was included as a dependent variable and baseline features as predictors in logistic regression analysis. This revealed no significant difference between participants with complete data and those with one or more missing data points (all *p* > 0.005). Pooled results of five multiple imputed datasets are presented. Only the outcome categories were calculated for completers.

The effect of CBT-E on each outcome measure (z-BMI, EDE-Q global and subscale scores, BSI and CIA global scores) over time was assessed via mixed-effects modeling of EOT and 20-week follow-up data. Within this model, the time variable was taken as fixed and the patients as random, with the assumption that they had been sampled at random from the same population, namely adolescents seeking treatment for anorexia nervosa. A two-level mixed-effects model with maximum likelihood estimation was used to analyze the data [29]. The model featured time nested within individuals [30,31].

To evaluate the role of DWS as a potential predictor of drop-out and/or outcomes at EOT and 20-week follow-up, stepwise logistic and linear regression analyses were performed. Confounding variables included in the model were: age, illness duration, and z-BMI, EDE-Q, BSI, and CIA global scores recorded at baseline. The variance inflation factor (VIF) was used to detect multicollinearity; a VIF >5 indicates the presence of numerical issues among the independent variables in a multivariate regression model [32].

All statistical analyses were carried out using SPSS software (IBM SPSS Statistics, version 28.0).

## 3. Results

### 3.1. The Sample

Characteristics of the 128 adolescent patients (128 females and 2 males) are given in Table 1. At their highest premorbid weight, the patients had a higher value than the population average, as evidenced by a z-BMI above zero (*p* < 0.001). The duration of their disease at admission was, on average, two years. All EDE-Q subscale and global scores were high, particularly “shape concern”.

### 3.2. Intent-to-Treat Findings at EOT and at a 20-Week Follow-Up

#### 3.2.1. Treatment Completion and Follow-Up

One hundred and seven patients (83.4%) completed the treatment (16.6% dropped out) and showed both considerable weight gain and reduced scores for eating-disorder and general psychopathology. There were no significantly different characteristics between the completers and non-completers at baseline.

Among completers, 72.9% completed the 20-week follow-up. About 94% of the participants received some form of post-discharge treatment, with 93.1% attending a 20-week CBT-E-based outpatient treatment delivered by trained therapists living close to their place of residence.

#### 3.2.2. Response to Treatment

Table 2 shows patients’ z-BMI, EDE-Q subscale and global scores, and BSI and CIA global scores at baseline, end-of-treatment, and at a 20-week follow-up, computed using the multiple imputation procedure. The z-BMI increased from baseline to end-of-treatment, remaining stable until the 20-week follow-up. Scores for eating-disorder and general psychopathology and clinical impairment fell significantly during therapy, results that were maintained at 20 weeks from the end-of-treatment. Weight and shape concern decreased from baseline to EOT and continued to decline from EOT to the 20-week follow-up.

The trajectories of change over time showed similar results among variables. Specifically, linear mixed models indicated an initial significant increase in z-BMI (linear growth) over treatment, and a subsequent deceleration in the rate of change (quadratic growth) during the follow-up. Both EDE-Q subscale and global scores (except EDE-Q subscales “weight concern” and “shape concern”) and BSI and CIA global scores mirrored this pattern, all significantly decreasing over the treatment and then displaying a deceleration in the rate of change up to the 20-week follow-up (Table 2).

Considering the outcome categories, 101 (94.4%) of the 107 patients who completed the treatment were classed as having a “good BMI outcome”, and 75 (70.1%) displayed a “full response” at the end-of-treatment. Among the 78 patients who completed the 20-week follow-up, 79.5% had a “good BMI outcome” and 61.5% had a “full response”.

### 3.3. Developmental Weight Suppression as a Predictor of Clinical Outcome

Logistic regression showed that DWS was not related to either drop-out, “good BMI outcome” or “full response” at either the EOT or at the 20-week follow-up (all *p* > 0.05).

However, linear regression analysis indicated that DWS was negatively correlated with EOT and the 20-week follow-up z-BMIs (beta = −0.28, t = −3.09, *p* = 0.003; beta = −0.38, t = −3.33, *p* = 0.001, respectively), with higher DWS being associated with lower BMI at EOT and the follow-up. No other associations were found (Table S1). In this statistical model, the baseline variables included as confounders were age, duration of illness, and EDEQ, BSI, and CIA global scores. The z-BMI was excluded because of the high multicollinearity (VIF > 10).

## 4. Discussion

This study aimed to evaluate the role of weight suppression in influencing drop-out and intensive CBT-E outcomes in adolescent patients with anorexia nervosa at EOT and a 20-week follow-up. The study had three main findings. The first was that 83.4% of patients completed the treatment. This percentage is in line with those found in our previous study [33], and similar to those from a randomized clinical trial on intensive CBT-E [34].

The second finding was related to the treatment outcome. Specifically, adolescent patients treated with intensive CBT-E achieved a substantial increase in weight, together with a marked decrease in eating-disorder and general psychopathology and clinical impairment scores. These improvements were either maintained or continued (as in “weight” and “shape concern”) at the follow-up, as demonstrated by the trajectories of change over time in the linear mixed models. Moreover, at EOT and the 20-week follow-up, almost 95 and 80% had a healthy BMI, and 70 and 60% also had minimal psychopathology, respectively. These results are similar to those found previously [33]. However, the adolescent patients in the present study achieved a higher percentage of remission at EOT than those treated as part of an RCT previously conducted in our clinic [35]. This finding could be attributed to the continuous improvement of CBT-E strategies and procedures implemented in our unit.

The third finding is new. Specifically, weight suppression negatively predicted BMI at EOT and follow-up, irrespective of other baseline clinical variables. In other words, the lower the baseline weight suppression, the better the outcome. This finding contrasts with those previously reported for two studies on adult patients with anorexia nervosa, which found that higher levels of weight suppression predicted greater total and faster rates of weight gain over the course of inpatient behavioral treatment [6] and outpatient treatment [4]. They also contrast with the non-treatment outcome study by Witt et al. [9], which found that baseline weight suppression was positively associated with BMI at 6- and 10-year follow-ups amongst individuals with adolescent-onset anorexia nervosa, identified through community-based screening. However, our data are partially in line with those found in a sample of adolescents with restrictive eating disorders treated via family-based treatment, in which lower weight suppression at intake was related to better outcomes at a 12-month follow-up, as defined by the absence of clinically significant ED psychopathology [11]. The discrepancies among the above studies could, however, be attributed to the different ages of patients evaluated in the treatment-outcome studies’ samples (adults vs. adolescents) and the treatment modalities implemented or not. Since higher weight suppression could correspond to a lower baseline BMI and higher maximum weight, we speculate that the negative association between weight suppression and BMI at EOT and follow-up found in our intensive CBT-E population could depend in part on the difficulties of adolescent patients with anorexia nervosa in accepting a healthy body weight.

To our knowledge, this is the first study to investigate the relationship between weight suppression and clinical outcomes in adolescent patients with anorexia nervosa treated via an evidence-based treatment. The main strength of the study is that the findings are likely to be both robust and generalizable because the cohort was sizeable and representative, being recruited from an Italian National Health System inpatient unit. Furthermore, we used a new method to calculate weight suppression (the DWS); this considers an individual’s developmental status at the highest premorbid weight in relation to their age, height, and sex. This method should come closer to accurately capturing the construct of weight suppression as it was originally developed [36]. Nevertheless, the study has some limitations. In particular, self-reporting of maximum weight may result in an incorrect premorbid z-BMI. In addition, we mainly used height at diagnosis as the height at maximal historical weight, which was rarely documented in the patient’s chart.

## 5. Conclusions

Our findings indicate that weight suppression is a negative predictor of BMI outcome in adolescent patients treated with intensive CBT-E. They also confirm that this treatment is suitable for adolescent patients with anorexia nervosa seeking treatment in a real-world clinical setting, being well-accepted and having a relatively low drop-out rate. In addition, they show a significant and stable increase in body weight (at least in the short-term) and a reduction in eating-disorder and general psychopathology and clinical impairment scores. Future research should confirm these findings, which potentially pave the way to using information about weight suppression to refine treatment and set individualized goals in adolescent patients with anorexia nervosa. Furthermore, the collection of biomedical parameters could furnish more information in this population.

## Figures and Tables

**Table 1:** Baseline characteristics of the 128 adolescents with anorexia nervosa.

Age at Presentation (Years)	16.8 ± 1.5 (Range 14–19)
z-BMI at presentation	−4.01 ± 2.27 (range −14.17–−0.85)
Age at top weight (years)	14.7 ± 1.7 (range 8–18)
z-BMI at top weight	0.21 ± 0.9 (range −2.34–2.22)
Duration of ED (years)	2.2 ± 1.8 (range 0–8)
Developmental weight suppression	4.2 ± 2.3 (range 0.37–14.11)
EDE-Q global score	3.9 ± 1.4
EDE-Q restraint	3.9 ± 1.7
EDE-Q eating concern	3.3 ± 1.4
EDE-Q weight concern	3.9 ± 1.6
EDE-Q shape concern	4.6 ± 1.5
BSI global score	1.1 ± 0.8
CIA global score	33.8 ± 11.0

A global EDE-Q score < 2.77 is comparable to the standard population. A global CIA score ≤ 16 indicates absence of clinical impairment.

**Table 2:** Pooled mean, standard error (SE) of baseline, end-of-treatment (EOT), and 20-week follow-up data of 128 adolescent patients with anorexia nervosa. Intent-to-treat analysis with multiple imputation procedure.

	Mean and (SE)			Analysis of Variance for Repeated Measures	Linear Mixed Model	
	Baseline ^a^	End-of-Treatment ^b^	20-Week Follow-Up ^c^	Pairwise Comparisons *	Linear Growth	Quadratic Growth
z-BMI	−4.01 (0.20)	−0.43 (0.06)	−0.97 (0.45)	a < b,c	β = 13.26, t = 9.53, *p* < 0.001	β = −11.37, *t* = −5.56, *p* < 0.001
Eating Disorder Examination Questionnaire						
Restraint	4.0 (0.2)	0.9 (0.2)	1.1 (0.5)	a < b,c	β = −10.4, *t* = −6.15, *p* < 0.001	β = 8.5, *t* = 4.19, *p* = 0.001
Eating concern	3.3 (0.1)	1.4 (0.1)	1.5 (0.1)	a < b,c	β = −6.9, *t* = −12.56, *p* = 0.001	β = 5.7, *t* = 8.67, *p* = < 0.001
Weight concern	3.9 (0.1)	2.0 (0.1)	1.7 (0.1)	a < b< c	β = −2.7, *t* = −12.39, *p* = 0.001	---
Shape concern	4.6 (0.1)	3.3 (0.1)	2.7 (0.2)	a < b<c	β = −2.2, *t* = −9.78, *p* = 0.001	---
Global score	3.9 (0.1)	1.9 (0.1)	1.7 (0.1)	a < b,c	β = −6.9, *t* = −12.0, *p* = 0.001	β = 5.1, *t* = 7.86, *p* < 0.001
Brief Symptom Inventory						
Global score	2.0 (0.1)	1.1 (0.1)	1.1 (0.1)	a < b,c	β = −3.4, *t* = −9.49, *p* = 0.001	β = 2.8, *t* = 7.07, *p* = <0.001
Clinical Impairment Assessment						
Global score	33.8 (1.0)	16.4 (1.4)	15.5 (1.9)	a < b,c	β = −62.7, *t* = −10.4, *p* = 0.001	β = 48.0, *t* = 6.55, *p* < 0.001

* Indicate significant differences (*p* < 0.05) between baseline (a), end-of-treatment (b) and 20-week follow-up (c).

## Data Availability

The data presented in this study are available on request from the corresponding author. The data are not publicly available due to privacy issues.

## Supplementary Materials

The following supporting information can be downloaded at: https://www.mdpi.com/article/10.3390/ijerph20043221/s1, Table S1. Logistic and linear regression analysis.
