# Supplementary material for: The Role of Weight Suppression in Intensive Enhanced Cognitive Behavioral Therapy for Adolescents with Anorexia Nervosa: A Longitudinal Study
Source: Int J Environ Res Public Health. 2023 Feb 12;20(4):3221. doi: 10.3390/ijerph20043221 (PMC9967636; doi:10.3390/ijerph20043221)
Supplement: Supplementary file 1 [file ijerph-20-03221-s001.zip › ijerph-2175870-supplementary.pdf]

Table S1. Logistic and linear regression analysis.

**Logistic regression analysis**			
**Dependent variable: drop-out**			
	OR (95%CI)	Wald test	p
DSW	1.04 (0.80-1.35)	0.09	0.767
Age	0.96 (0.65-1.44)	0.03	0.859
Duration of illness	0.87 (0.60-1.27)	0.50	0.480
EDE-Q global score	1.32 (0.74-2.36)	0.86	0.353
BSI global score	1.63 (0.59-4.50)	0.89	0.345
CIA global score	0.90 (0.82-0.99)	4.39	0.036
**Dependent variable: "good BMI outcome" at end of treatment**			
	OR (95%CI)	Wald test	p
DSW	0.84 (0.62-1.14)	1.26	0.262
Age	0.75 (0.35-1.60)	0.56	0.453
Duration of illness	0.73 (0.44-1.22)	1.40	0.236
EDE-Q global score	2.71 (1.07-6.84)	4.45	0.035
BSI global score	0.31 (0.05-1.78)	1.73	0.189
CIA global score	1.03 (0.89-1.20)	0.16	0.685
**Dependent variable: "full response" at end of treatment**			
	OR (95%CI)	Wald test	p
DSW	0.77 (0.63-1.79)	2.03	0.170
Age	0.97 (0.70-1.36)	0.02	0.880
Duration of illness	1.07 (0.81-1.41)	0.20	0.653
EDE-Q global score	0.59 (0.35-0.98)	4.14	0.042
BSI global score	0.73 (0.29-1.85)	0.43	0.511
CIA global score	1.04 (0.96-1.14)	1.04	0.308
**Dependent variable: "good BMI outcome" at 20-week follow-up**			
	OR (95%CI)	Wald test	p
DSW	0.79 (0.62-1.03)	3.12	0.077
Age	0.92 (0.58-1.47)	0.11	0.740
Duration of illness	1.30 (0.83-2.01)	1.32	0.250
EDE-Q global score	0.75 (0.37-1.50)	0.66	0.415
BSI global score	0.53 (0.14-2.04)	0.85	0.358
CIA global score	1.13 (1.00-1.28)	3.64	0.057
**Dependent variable: "full response" at 20-week follow-up**			
	OR (95%CI)	Wald test	p
DSW	0.90 (0.72-1.12)	0.88	0.349
Age	0.96 (0.67-1.38)	0.04	0.835
Duration of illness	1.20 (0.88-1.65)	1.33	0.248
EDE-Q global score	0.77 (0.43-1.39)	0.74	0.388
BSI global score	0.37 (0.12-1.16)	2.89	0.089
CIA global score	1.10 (1.00-1.22)	3.59	0.058
**Linear regression analysis**			
**Dependent variable: z-BMI at end of treatment**			
	beta	t	p
DSW	-0.28	-3.09	0.003
Age	-0.50	-5.57	<0.001
Duration of illness	0.12	1.31	0.194
EDE-Q global score	0.23	1.83	0.070
BSI global score	-0.08	-0.58	0.563
CIA global score	-0.06	-0.41	0.682
**Dependent variable: z-BMI at 20-week follow-up**			
	beta	t	p

DSW	-0.38	-3.33	0.001
Age	-0.05	-0.45	0.653
Duration of illness	0.17	1.39	0.170
EDE-Q global score	-0.23	-1.35	0.182
BSI global score	0.03	0.15	0.881
CIA global score	0.30	1.31	0.194
**Dependent variable: EDE-Q global score at end of treatment**			
	beta	t	p
DSW	0.02	0.18	0.858
Age	-0.05	-0.53	0.595
Duration of illness	-0.17	-1.92	0.058
EDE-Q global score	0.49	3.96	<0.001
BSI global score	0.08	0.53	0.594
CIA global score	0.06	0.406	0.686
**Dependent variable: EDE-Q global score at 20-week follow-up**			
	beta	t	p
DSW	0.06	0.49	0.628
Age	-0.10	-0.84	0.401
Duration of illness	-0.10	-0.83	0.407
EDE-Q global score	0.28	1.61	0.111
BSI global score	0.24	1.20	0.234
CIA global score	-0.09	-0.40	0.687
